# Hydration Status, Dietary Habits, and Functional Food Consumption Preferences of Football Athletes: A Cross-Sectional Pilot Study

**DOI:** 10.3390/nu17061078

**Published:** 2025-03-19

**Authors:** Georgios Papaoikonomou, Aikaterini Kandyliari, Antonis Vlassopoulos, Olga Malisova, Antonios E. Koutelidakis

**Affiliations:** 1 Department of Food Science and Technology, University of Patras, G Seferi 2, 30100 Agrinio, Greece; g_papaoikonomou@upatras.gr; 2 Laboratory of Nutrition and Public Health, Department of Food Science and Nutrition, University of the Aegean, Dimokratias 66, 81400 Lemnos, Greece; kkandyliari@aua.gr (A.K.); akoutel@aegean.gr (A.E.K.); 3 Food Science and Human Nutrition, Agricultural University of Athens, 11855 Athens, Greece; avlassopoulos@aua.gr

**Keywords:** hydration, hydration indices, athletes, dietary habits, functional food, hydration knowledge

## Abstract

**Background/Objectives**: Hydration and nutrition are two key aspects of high-quality athletic performance. However, little is known about the potential beneficial effects of functional foods in sports. The present study investigates the hydration statuses of and knowledge, dietary habits, and consumption of functional foods among football athletes, both professional (*n* = 24) and non-professional (*n* = 20). **Methods**: The study sample had a mean age of 19.9 ± 4.9 years, a mean weight of 74.0 ± 6.0 kg, and a mean body mass index (BMI) of 23.0 ± 1.40 km/m^2^. All the athletes filled out a questionnaire about their hydration knowledge regarding sports and a functional food consumption questionnaire. Hydration status was assessed through urine color (Ucol) before and after training. **Results**: The results of this study show that 65% of the non-professional and 59.1% of the professional football players started their training dehydrated, and this proportion increased to 73.7% at the end of the training. Moreover, >50% of athletes were poorly educated in hydration practices during and after exercise. In addition, nutritional habits differences were observed among the professional and non-professional athletes regarding alcohol consumption (*p* < 0.0001) and fries (*p* < 0.05). **Conclusions**: A comprehensive understanding of and tailored approaches to nutrition and hydration need to be promoted among football athletes to realize the benefits of hydration and nutritional strategies that optimize their physiological resilience and competitive edges.

## 1. Introduction

Proper hydration in sports is necessary for optimal muscular system performance [1,2]. All humans lose body fluids daily through breath, urine, and feces, especially athletes like football players, who also need to deal with extensive sweat loss during exercise [3]. Therefore, many hydration plans have been proposed for the achievement of a proper hydration status [4,5,6]. The primary aim of hydration practices in regard to football athletes is to optimize fluid balance, ensuring that they are adequately hydrated before, during, and after training sessions and matches [7,8]. This involves a delicate equilibrium between fluid intake and loss, considering factors like sweat rate, environmental conditions, and individual physiological variations. By understanding and implementing tailored hydration strategies, football athletes can enhance their endurance, cognitive function, and overall well-being, thereby maximizing their potential on the field [9].

Achieving and maintaining an optimal water balance is therefore of paramount importance for football athletes. Athletes whose sweat exceeds their fluid replacement levels during exercise become dehydrated. Dehydration equivalent to 3% or more of an athlete’s body weight disrupts physiological function and increases their risk of developing a heat-related illness, heat stroke, or heat cramps [1]. This is especially true for football, which is characterized by intermittent bursts of high-intensity activity, prolonged matches, and diverse weather conditions, placing a substantial strain on the players’ bodies [10]. Hence, effective hydration practices are integral not only for sustaining peak performance but also for protecting athletes from the detrimental effects of dehydration, such as impaired cognitive function, decreased endurance, and increased susceptibility to injuries [11]. The process of thermal acclimatization should be considered when formulating individualized rehydration strategies. However, it is important to note that universally applicable strategies cannot be derived given the variability in sweating rates between athletes [6]. According to training models and protocols of professional athletes, it seems that a drink comprising 6–8% carbohydrates and electrolytes can maintain athletic performance [12], while in physically active athletes, a balanced diet is adequate for exercise [6].

Proper hydration knowledge is a cornerstone in the arsenal of tools for proper hydration, enhancing the athletic prowess and overall well-being of football athletes, given the rigorous physical demands and environmental factors inherent in this sport [13]. However, poor hydration knowledge is commonly observed among athletes [14,15], and therefore understanding optimal hydration practices is pivotal. Research has shown that athletes who are well informed about hydration strategies showed better results on questionnaires regarding sport hydration knowledge [16]. 

On the other hand, the energy balance of athletes is another key aspect for achieving optimal athletic performance; athletes, including football players, must receive adequate amounts of macronutrients and micronutrients through the consumption of a wide variety of foods [17]. A sufficient qualitive nutrition threshold should also be in place to facilitate the maintenance of peak performance levels throughout the course of a game [18]. For instance, during high-intensity exercise, such as the type engaged in by football players, the daily carbohydrate intake is recommended to reach 6–10 g/kg of one’s bodyweight (BW), while protein intake should be around 1.2–2.0 g/kg of BW/d to retain fat-free-mass, and all micronutrients should be consumed to maintain mental and body performance [19]. However, in many cases, professional players do not seem to consume adequate amounts of food for energy and, as a result, do not achieve the energy recommendations set by the American College of Sports Medicine (ACSM) [20].

As football is a physically demanding sport, apart from energy, it requires a harmonious blend of macronutrients, micronutrients, and bioactive compounds such as antioxidants to sustain energy levels, support muscle recovery, and have a physiological impact [21]. Although exercise has many positive effects on the human body, it may induce unpleasant changes in physiology, which are associated with malfunctions in the immune system, increased levels of inflammation, and oxidative stress [22]. During exercise, oxygen consumption within the muscles may increase up to 15 times, which may lead to the overproduction of free radicals, also known as reactive oxygen species (ROS), in the human body [23]. Therefore, functional foods and drinks enriched with specific nutrients and bioactive ingredients offer a targeted approach to addressing the unique nutritional needs of football athletes [24]. In detail, research has shown that some ingredients, including carotenoids, flavonoids, probiotics, minerals, and vitamins, are associated with potential antioxidant and anti-inflammatory activity [25], and therefore functional drinks such as beetroot juice can strengthen the neuromuscular system between short, repeated sprints [26], as engaged in by football players on the field. Other sport drinks containing almond oils, sodium, potassium, α-tocopherol, and polyphenols also have scientifically proven anti-inflammatory properties [27]. Thus, whether functional foods act by promoting a faster recovery through anti-inflammatory properties or bolstering endurance with energy-dense components, their strategic incorporation into an athlete’s diet can have a profound impact on their overall performance [28]. 

Based on the above, the primary aim of this study is to unravel the nuanced intricacies of hydration practices within a footballing community of professional and non-professional football athletes by shedding light on their dietary preferences and functional food choices. In particular, we aim to evaluate and compare hydration levels and highlight potential differences between professional and amateur athletes through the study of hydration indicators and their hydration knowledge. In addition, we aim to investigate their dietary habits regarding the consumption of different food and functional food products. Through this investigation, we seek to provide valuable insights that can inform tailored strategies for enhancing the overall well-being and performance of football athletes by demonstrating a measurable correlation between their specific hydration practices, dietary habits, and functional food choices and their respective performance levels, with a particular focus on identifying significant differences between professional and amateur players. 

This study was carried out to test the hypothesis that professional football athletes, due to their specialized training and support, would present with superior hydration statuses, superior hydration knowledge, and more optimized dietary behaviors compared to non-professional athletes

## 2. Materials and Methods

### 2.1. Participants

The study sample consisted of forty-four athletes, including professional (*n* = 24) and non-professional (*n* = 20) football players from two different sport clubs, respectively. A notable distinction between professionals and non-professional football players emerged in terms of training frequency and associated remuneration. Professionals engage in training more frequently, e.g., more than five times per week, than non-professionals.

All participant athletes enrolled in this study were male and lived in Thessaloniki, Greece. Τhe doctors for each group confirmed that all participants were healthy and did not have any impending diseases, constituting the main criterion for inclusion in this study. This study was carried out in the athletes’ training areas (field areas), and for the collection of urine and fulfillment of questionnaires, a room with sufficient lighting was provided.

### 2.2. Study Protocol 

The study protocol was approved by the Research Ethics Committee of the Aegean University under protocol number 31672/26.09.2023.

Prior to performing this study, each athlete who wanted to participate in the study was informed about his rights regarding participation in this research, in accordance with the Convention on Human Rights and Bioethics. All participants were informed about the aims and methodology of this study, both orally and in written form, and all questions were answered. Each participant then signed a written form stating that they declared that they had broadly been informed about the study and understood the proposed procedure and that all their personal data were confidential. The research team agreed to provide their professional knowledge in accordance with the protocol, the provisions of the Declaration of Helsinki, and the provisions of the General Data Protection Regulation. 

All data collection steps, such as urine sample analysis and pH determination, and the study results concerning dietary habits were kept locked in the office of the principal investigator in the University of the Aegean in Lemnos. Also, personal data such as age, gender, weight, and educational level were kept confidential in accordance with the Convention on Human Rights and Bioethics (2619/1998).

### 2.3. Questionnaires 

All participants filled in a hydration knowledge questionnaire (HKQ) [14] and a validated functional food frequency questionnaire (FFFQ) [29] so we could evaluate their food and hydration habits, attitudes, and knowledge in relation to their sport activity. 

FFFQ consisted of questions on frequency of consumption of specific functional food products, and the frequency of consumption was recorded as ‘everyday’, ‘3–6 times per week’, ‘2 times per week’, ‘once a week’, ‘1–2 times per month’, and ‘seldom/never’.

HKQ consisted of 17 “true” or “false” questions. At the end of each questionnaire, a score was given. The score was calculated by adding the total number of correctly answered questions. The minimum score that could be obtained was 0 (0% correct answers), and the highest score was 17 (100% correct answers). 

All questionnaires were completed in approximately 30 min. 

### 2.4. Urine Sample Collection and Analysis

Each participant was provided with two sterile plastic urine collection containers and asked to collect one urine sample prior to and one after each training session. Each training session lasted one hour and was carried out between 18:00 and 20:00 on a weekday. The mean temperature recorded in both studies was 17ο Celsius. Urine samples were collected by the athletes themselves and kept in a box with ice packs until given to the research team. 

More specifically, from the non-professional team (*n* = 20 athletes in total), 20 athletes submitted urine samples before training; by the end of training, two athletes could not complete the procedure (*n* = 18). From the professional team (*n* = 24 athletes in total), 22 athletes submitted urine samples before training, while at the end of the training, 4 athletes did not manage to complete the procedure (*n* = 20).

Participants delivered all urine samples to the research team. Ucol and pH analyses were then conducted to determine the color of urine before and after training using a specific color chart (with a scale from 1 to 8) at Dictionary of Color, Maertz and Paul, 2nd edition, 1950 (Armstrong et al. 1994) [1]. The urine samples were characterized by their level of hydration, with a score of 1–3 for well hydrated, 4–5 for moderately dehydration, and 6–8 for serious dehydration. In addition, the pH of the urine samples was determined with pH color fixed indicator strips (Macherey-Nagel, Dueren, Germany). 

### 2.5. Statistical Analysis

Data analysis was conducted using SPSS (version 19), (SPSS, Inc., Chicago, IL, USA). The Kolmogorov–Smirnov test was used to assess normality. The Mann–Whitney test and *t*-test were used to determine statistically significant differences between variables, and the Two-Sample Independent *t*-test was used to determine the differences between samples regarding color and pH. For categorical data with expected frequencies < 5, Fisher’s exact test was used. A post hoc power analysis, based on a sample size of 24 professional and 20 amateur soccer players, an alpha of 0.05, and a medium effect size, revealed a statistical power of 0.365. The level of significance was set at 0.05.

## 3. Results

### 3.1. Demographic and Anthropometric Characteristics of Professional and Non-Professional Athlete Participants

As presented in Table 1, the participants in this study were all male and had a mean age of 19.90 ± 4.9 years, and the mean BMI was 23.0 ± 1.4 kg/m^2^. The non-professional team consisted of 20 athletes, while the professional team consisted of 24 athletes. The athletes on the professional team were younger, with a mean age of 17.60 ± 0.10 years (*p* < 0.001), and had engaged in fewer years of study (11.50 ± 0.50 years, *p* < 0.001) compared to the athletes on the non-professional team.

### 3.2. Hydration Status Determination

As shown in Table 2, Ucol and pH data were collected and characterized according to the special color chart (1–8) for both the non-professional and professional athletes before and after training. Before training, the non-professional athletes were given a mean Ucol of 3.9 ± 2.1, while that for the professional athletes was 4.0 ± 1.2, indicating that both athletic groups started the training in a dehydrated condition. In addition, after training, the athletes were also characterized as dehydrated, with mean Ucol values of 4.4 ± 2.1 and 4.8 ± 1.1 for non-professional and professional athletes, respectively. No significant statistical differences were found between the professional and non-professional football players both pre- and post-training. 

The mean Ucol for the entire sample (*n* = 44) was 3.9 ± 1.7. At the end of the training, the mean Ucol increased to 4.6 ± 1.6, indicating that the average profile of the total sample exhibited signs of dehydration. Regarding the pH of the urine samples before training and after training, a statistically important decrease was observed for the non-professionals, with values of 6.1 ± 1.2 and 5.1 ± 0.3 after training. The professionals did not present a statistically important difference.

As indicated in Table 3, 38.1% of the athletes began the training period with adequate hydration levels. However, by the end of the training period, 73.7% of the football players exhibited symptoms of dehydration. Furthermore, 25% of the professional football players concluded the training period in a state of severe dehydration, while the percentage of non-professional players who finished the training period in a similar state was even higher (38.9%).

### 3.3. Hydration Knowledge

The mean hydration knowledge score from the hydration knowledge questionnaire (HKQ) for the total number of athletes was 8.0 ± 3.10. The non-professional football players had better hydration knowledge scores than the professional football players, with 9.0 ± 2.80 and 7.30 ± 3.30, respectively. Therefore, both groups showed low hydration knowledge with respect to the 17 questions regarding hydration practices in sports.

Specifically, as presented in Table 4, 33 athletes (75%) responded that thirst being the best indicator for dehydration was false, and 37 athletes (84.1%) responded that the claim that dehydration decreases athletic performance is true. In addition, 34 athletes (77.3%) did not respond correctly to the question of whether an athlete should drink sports drinks rather than water when exercising for more than one hour. A significant difference was found between the non-professional and professional football athletes in the responses to the questions of whether thirst is the best indicator of hydration (*p* < 0.044) and whether coaches should not allow fluid consumption during exercise (*p* < 0.002).

### 3.4. Hydration Habits

As presented in Table 5, all the athletes (*n* = 44) responded to the questions regarding the consumption of soft drinks and alcoholic beverages on a weekly basis. Professional football athletes seem to avoid frequent consumption of soft drinks, wine, beer, and other alcohol drinks; in contrast, the non-professionals have a balanced allocation. In addition, 60% of the non-professional athletes consume soft drinks more than 2 days per week. In contrast, 62.5% of the professionals do not seem to prefer this type of drink. 

Almost all the professional athletes avoided consuming alcoholic drinks. Moreover, the professional athletes have a more balanced allocation of energy drinks and fruit juices compared to the non-professionals. A significant difference was found regarding fruit juice consumption between the non-professional and professional athletes (*p* = 0.025). Moreover, a statistically significant difference was found in relation to beer and alcohol consumption (*p* < 0.0001 and *p* < 0.001, respectively). 

### 3.5. Frequency of Consumption of Functional Foods and General Dietary Habits

As shown in Table 6, all the football players (*n* = 44) completed a Food Frequency Questionnaire (FFQ). Non-professional and professional athletes are labeled as N and P, respectively. It was observed that the professional football players followed a more balanced diet than the non-professional football players. The non-professional football athletes (50%) consume potato chips and fries 1–2 times per week. In contrast, only 4.2% of the professional football players consume this type of food. In addition, the consumption of meats, cereals, eggs, and spaghetti in both groups seems to be more balanced.

The chi-square x^2^ test was used to find differences in dietary habits between professional and non-professional football athletes. A significant difference was found in beef consumption (*p* = 0.007). The professional football players (63%) consume beef 1–2 times a week; in contrast, the non-professional players consume it 1–3 times a month (40%). Moreover, significant differences were found in the consumption of fries (*p* < 0.001), as professionals (83%) declared that they consume fries 1–3 times a month; in contrast, the non-professional athletes (50%) stated they consumed them 1–2 times per week. In addition, significant differences were found in the consumption of dried fruits (*p* < 0.01) and potato chips (*p* < 0.001). Finally, significant differences were found in the consumption of oil (*p* = 0.021), with professionals (42%) reporting oil consumption 1-2 days per week.

As shown in Table 7, all the football players (*n* = 44) completed a frequency of functional food consumption questionnaire (FFFQ). Many functional foods, such as spirulina, soy, turmeric, crocus sativus, propolis, and probiotics, are consumed in low amounts. In contrast, dairy products, honey, fruits, and vegetables have more balanced allocation in the athletes’ diets.

Chi-square x^2^ analysis was used (with the results shown in Table 7) to find statistically important differences between non-professional and professional football athletes in terms of functional food consumption. A significant difference was found in the consumption of energy drinks (*p* = 0.017) and coffee (*p* = 0.021). Statistically significant differences were also found in egg consumption (*p* = 0.026). In contrast to non-professional athletes, professional football players seem to prefer to consume eggs once per week. 

## 4. Discussion

### 4.1. Fluid Balance

The findings of this study indicate that both groups began and completed their training in a state of dehydration, with percentages of 69.1% and 73.7%, respectively. This observation is consistent with the findings of numerous studies that have documented the prevalence of dehydration among young athletes during training [30,31]. Accordingly, in a study by Webb et al. [32], 52 athletes’ mean pre–post Ucol scores were 4.31 ± 1.75 and 5.67 ± 1.45, respectively, constituting analogous findings. Additionally, in a study involving 25 soccer athletes with a mean age of 22.3 ± 1.1 years, Ucol and urine specific gravity (USG) assessments were conducted for 3 days during exercise. The analyses revealed that the USG score was 1.025 ± 0.007 on the first day, 1.019 ± 0.010 on the second day, and 1.022 ± 0.009 on the third day. Additionally, the Ucol scores were recorded as 4 ± 1, 3 ± 1, and 4 ± 1, respectively [33]. Moreover, in a study involving 16 adolescents (16 ± 4.5 years) with a mean weight of 51 ± 24.8 kg, the results of a pre-exercise exercise analysis showed that based on a urine color scale, 68.8% of the athletes were hydrated (1–3), and only 31.3% were dehydrated according to the color scale (4–5) [34]. Post-exercise analysis showed that 37.5% remained hydrated, and 43.8% of the soccer athletes were dehydrated, with a score of 4–5, while 18.8% finished their training seriously dehydrated, with a color score of 6–8 according to the special color chart. Moreover, in another study, involving 59 adolescent athletes from different sports (15.2 ± 1.3 years), the mean pre–post training Ucol values of all athletes were 4 ± 1 and 5 ± 1 in the early hours, and changes in body weight were observed, with a score of −1.1 ± 0.07% [35]. It is evident that hydration practices and environmental factors must be considered when addressing issues related to excessive sweating [4]. Intense sweating during physical activity can lead to significant weight loss, resulting in increased fluid replenishment requirements. The variability in sweat rates [36], ranging from 0.5 to 1.9 L/h, emphasizes the importance of hydration strategies on an induvial level [37]. Despite acquiring adequate hydration during practice sessions to prevent exacerbation of pre-practice hypohydration, adolescent participants exhibited inadequate hydration between practices, resulting in mild hypohydration [31], as the majority of athletes (more than 50%) arrive at training already dehydrated [6,38].

### 4.2. Hydration Knowledge and Fluid Replacement

The current study reveals important findings regarding knowledge of hydration and fluid replacement, especially among athletes. The total scores of the total answers collected from non-professional athletes and professional athletes were 9.0 ± 2.8 and 7.30 ± 3.3, respectively, while the total mean score for the entire sample (*n* = 44) was 8.0 ± 3.1 for a total of 17 questions. Among the athletes, 75% answered positively that thirst is the best indicator for dehydration (4), while thirst is an indication that our body is already dehydrated. A total of 84.1% answered positively that dehydration reduces athletic performance, and 72.7% answered that the claim that sports drinks are better because they contain glucose was false. A similar study observed similar findings, with 68.3% of participants indicating that thirst is the most reliable indicator of dehydration. Furthermore, 85% of athletes responded positively to the question of whether dehydration has a negative impact on sporting performance, while 85% responded positively to the question of whether Ucol can be used as an effective indicator of dehydration [15]. In another study conducted by Nichols et al., 66.2% of athletes gave the correct answer to the question of whether weighing oneself before and after training is a reliable method for determining the amount of fluid that one needs to consume. Furthermore, 91.4% of the volunteers gave a positive answer to the question of whether dehydration has a negative impact on athletic performance. The mean hydration knowledge score was higher, at 13.9 ± 1.8. [14]. The results of the present study are similar to those of other studies, which also found moderate levels of hydration knowledge [39,40]. Given that athletes are more likely to lack basic knowledge in this regard, it is imperative to recognize the importance of better nutritional practices and better nutritional decisions [41]. A holistic approach should be employed, with education on hydration strategies, knowledge, and assessment beginning at an early age. It is widely accepted that adults who adhere to a healthier dietary pattern typically also have a healthier fluid pattern, characterized by increased water and total fluid consumption [42]. Further research is warranted to investigate the potential influence of misinformation, as the observed knowledge deficit may not be solely attributable to information scarcity.

### 4.3. Eating Habits and Functional Food

**Figure 1:**
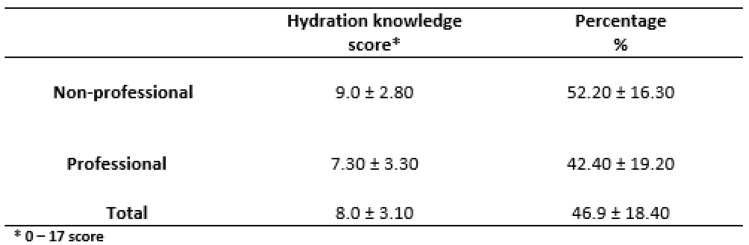
Total sample knowledge score from 17 questions about hydration knowledge and practices during training.

Regarding eating habits with respect to functional foods, the questionnaire contained food rich in bioactive components with potential benefits for chronic diseases such as diabetes, obesity, and metabolic syndrome but also potential benefits for injured muscular, recovery, and endurance systems [25]. Concerning consumption of functional food (Table 7), 20.5% of all the football athletes consume such food, with 11.4% consuming vegetables, 25.6% consuming energy drinks, 13.6% consuming coffee, and 28.2% consuming cereals 1–2 times per week. Significant differences were observed regarding eggs (Table 7) between the non-professional and professional football athletes as well as in terms of coffee and energy drinks. In a study of 88 American football players with a mean age of 19.6 ± 1.7 years, energy drinks were consumed only 1–2 times per week, and the consumption value for fruits was 24.4%, with 14.8% for coffee [43]. In another study, Smarkusz et al. included 44 American football athletes, with a mean age of 25.1 ± 5.8 years for defensive athletes and 23.4 ± 3.7 years for offensive athletes. A significant difference was identified in the consumption of energy drinks among offensive and defensive players (*p* < 0.05) [44]. We can assume that the consumption of a caffeinated energy drink gives a positive muscular and psychological boost to an athlete, as there are data showing that the consumption of an energy drink containing caffeine increases the subjective perception of muscular strength during exercise [45]. In an epidemiological study of selected functional foods conducted by Kandyliari et al., 949 volunteers were examined. The findings demonstrated a positive correlation between increased consumption of specific functional foods and reduced BMI, thereby corroborating the prevailing assumptions concerning the health-promoting properties of functional foods [46]. It should be noted that the chronic consumption of polyphenols, such as epicatechin and resveratrol, has been shown to have a positive effect on athletes’ performance. Similarly, the intake of vitamin E has been observed to have a beneficial effect on competitive athletes [47]. Moreover, it appears that low doses of quercetin can enhance the body’s response, resulting in increased muscular and mental performance, two critical aspects of playing football [48]. Concerning dietary patterns, a systematic review revealed an inadequate supply of energy, carbohydrates, vitamins, and minerals (including calcium, magnesium, and iron as well as iodine) in male football players [49]. Noronha et al. [50] observed that the more educated the athlete in nutrition practices, the better the score on nutrition assessment tests. Adolescents, who are often rich in talent, do not focus on nutrition and hydration strategies. As a result, individual athletic performance can be low, with issues such as dehydration and inadequate nutrition levels. For example, in the present research, the hydration knowledge score for the professionals was 42.40% ± 19.20% (Figure 1). Educational interventions concerning nutrition and hydration issues could improve this behavior. In a study by Trakman et al. [51], 41 adolescents with a mean age of 15.0 ± 0.4 years and a BMI of 19.1 ± 1.5 kg/m^2^ participated in an educational intervention for nutrition improvement. Adolescent athletes were asked to fill in questionnaires regarding nutrition four times: the first time was before the intervention; the second was after the intervention; the third was after 6 weeks; and the fourth was after 12 weeks [52]. A significant difference was found between the first and the last three answers given [52]. Thus, there is evidence that young athletes’ knowledge regarding nutrition and hydration in sports can be improved by educating them. To mitigate the potential for suboptimal dietary practices and associated performance limitations, a proactive educational approach is required. Engaging young athletes with evidence-based nutrition education, facilitated by experts in sports nutrition and dietetics, can promote the development of healthy eating habits and optimize physiological adaptations to training, ultimately contributing to long-term athletic success and well-being.

### 4.4. Limitations of This Study

We acknowledge several inherent methodological limitations. Firstly, the assessment of hydration status relied primarily on urine color analysis, which, while practical, is subject to individual variability and potential confounding factors. Future research should consider incorporating more objective measures, such as urine specific gravity or osmolality, to provide a more comprehensive and accurate evaluation of hydration levels. Secondly, we acknowledge that because this study was a pilot study, we conducted a post hoc power analysis for our sample size of 44 soccer players, resulting in a statistical power of 0.37, which is below the commonly recommended value of 0.8. However, despite this limitation, our study still yields statistically significant results, indicating a strong trend that warrants further investigation. This suggests that the observed effect may be meaningful and could be explored in larger cohorts to confirm its robustness. This study could be expanded to include a female sample. This would enhance the statistical power and generalizability of the findings, particularly in the context of inter-individual variations in hydration responses. Finally, the urine color analysis was conducted at a single time point (pre- and post-training). To improve the reliability and robustness of these assessments, future studies should implement repeated measurements across multiple training sessions, thereby capturing the dynamic fluctuations in hydration status associated with training load and environmental factors.

## 5. Conclusions

The aim of this study was to assess the dietary habits, functional food consumption habits, and hydration statuses of professional athletes and non-professional athletes. Hydration status was evaluated using urine color analysis (Ucol). The results revealed significant dehydration in both groups, corroborating previous findings that highlight a prevalent lack of hydration knowledge among football players. Specifically, the participants demonstrated a limited understanding of effective hydration strategies and behaviors, as evidenced by the low scores on the hydration knowledge questionnaire (HKQ). Consequently, there is a clear need for targeted educational interventions to improve hydration literacy in this population. Regarding the nutritional habits of the athletes, non-professionals presented a more careless diet compared to the professionals, as shown in Table 6. Nevertheless, all the football players presented a low consumption of functional foods (Table 5). Given the critical roles of hydration and nutrition in maintaining athletic performance and physiological homeostasis, these findings underscore the necessity of comprehensive interventions. Sports nutritionists and coaches should prioritize the development and implementation of evidence-based educational programs to address the identified knowledge gaps and promote optimal hydration and nutritional practices among football players. While the primary focus of this study is on football players and the related areas of knowledge gaps, evidence-based nutrition, and improved hydration strategies, the findings can be adapted in other sport disciplines to meet the demands of various events and sports. This includes endurance sports, high-intensity sports, and strength-based sports.

## Figures and Tables

**Table 1:** Demographic and anthropometric characteristics of the professional and non-professional athletes (mean ± SD).

	Non-Professional Athletes (*n* = 20)	Professional Athletes (*n* = 24)	Total Athletes (*n* = 44)	*p*-Value
Gender (%male)	100	100	100	
Age (y) *	22.7 ± 6.0	17.6 ± 0.1	19.9 ± 4.9	<0.001
Height (m)	1.8 ± 0.1	1.8 ± 0.1	1.8 ± 0.1	0.67
BMI (kg/m^2^)	23.3 ± 1.7	22.6 ± 1.0	23.0 ± 1.4	0.11
Weight (kg)	76.0 ± 6.0	73.0 ± 6.0	74.0 ± 6.0	0.15
Years of study (y) *	13.0 ± 2.0	11.5 ± 0.5	12.3 ± 1.3	<0.001
* Non-normal distribution according to Kolmogorov–Smirnov test analysis				

No statistically important differences were observed for height, weight, or body mass index (BMI) between non-professional and professional athletes.

**Table 2:** Pre- and post-training comparison of urinary indices of hydration status.

		Pre-Training	Post-Training	*p*-Value *
Non-professional athletes (*n* = 20)	Ucol	3.9 ± 2.1	4.4 ± 2.1	0.92
	Urine pH	6.1 ± 1.2	5.1 ± 0.3	<0.001
Professional athletes (*n* = 24)	Ucol	4.0 ± 1.2	4.8 ± 1.1	0.77
	Urine pH	6.8 ± 0.9	6.1 ± 0.8	0.78
Total athletes (*n* = 44)	Ucol	3.9 ± 1.7	4.6 ± 1.6	0.94
	Urine pH	6.5 ± 1.1	5.6 ± 0.8	0.03

* Differences between pre- and post- training for non-professional and professional athletes, determined using independent-samples *t*-test.

**Table 3:** Hydration status distribution of non-professional and professional football players.

		*n* (%)	
	Hydration Status According to Ucol Score *	Pre-Training	Post-Training
Non-professional athletes (*n* = 20)	Hydrated (1–3)	7 (35%)	7 (38.9%)
	Dehydrated (4–5)	6 (30%)	4 (22.2%)
	Severely dehydrated (6–8)	7 (35%)	7 (38.9%)
Professional athletes (*n* = 24)	Hydrated (1–3)	9 (40.9%)	3 (15%)
	Dehydrated (4–5)	10 (45.5%)	12 (60%)
	Severely dehydrated (6–8)	3 (13.6%)	5 (25%)
Total athletes (*n* = 44)	Hydrated (1–3)	16 (38.1%)	10 (26.3%)
	Dehydrated (4–5)	16 (38.1%)	16 (42.1%)
	Severely dehydrated (6–8)	10 (23.8%)	12 (31.6%)

* Ucol score between 1–8 [1].

**Table 4:** Responses of football players (N = 44) from the hydration knowledge questionnaire.

	Knowledge Questions Regarding Hydration and Fluid Replacement *	TRUE Ν (%)	FALSE Ν (%)
1	Salt tablets prevent dehydration during competition training. ^2^	22 (50%)	22 (50%)
2	Thirst is the best indicator of dehydration. ^2^	33 (75%)	11 (25%)
3	Dehydration decreases athletic performance. ^1^	37 (84.1%)	7 (15.9%)
4	Athletes should not drink water or fluids during practice. ^2^	5 (11.24%)	39 (88.6%)
5	Coaches should not let players drink fluids during practice. ^2^	35 (79.5%)	9 (20.5%)
6	Coaches should not let players drink fluids during competition. ^2^	26 (59.1%)	18 (40.9%)
7	It is important for fluids to be readily available to athletes during practice. ^1^	35 (79.5%)	9 (20.5%)
8	It is important for fluids to be readily available to athletes during competition. ^1^	34 (77.3%)	10 (22.7%)
9	Athletes should drink sports drinks within 2 h after exercise. ^1^	16 (36.4%)	28 (63.6%)
10	Sports drinks are better than water because they restore glycogen in muscles. ^1^	12 (27.3%)	32 (72.7%)
11	An athlete should drink 17–20 fl. oz. of water or sports drink a couple of hours before exercise ^1^	19 (43.2%)	25 (56.8%)
12	An athlete should drink 7–10 fl. oz. 10–20 min before competition. ^1^	17 (38.6%)	27 (61.4%)
13	When exercising more than one hour, an athlete should drink sports drinks rather than water. ^1^	10 (22.7%)	34 (77.3%)
14	Monitoring color of urine is a way an athlete can judge if he/she is dehydrated. ^1^	27 (61.4%)	17 (38.6%)
15	Weighing before and after practice is a good way to determine how much fluid to consume. ^1^	19 (43.2%)	25 (56.8%)
16	Excessive sweating, thirst, and cramping are signs of dehydration. ^1^	25 (56.8%)	19 (43.2%)
17	More than 2 alcoholic drinks the day before competition can lead to dehydration. ^1^	21 (47.7%)	23 (52.3%)

* (^1^) indicates originally true answers, and (^2^) denotes originally false answers.

**Table 5:** Percentages of consumption of soft drinks and alcoholic beverages between non-professional and professional football players.

%	Never/ Rarely	1–2 Week	3–6 Week	1–2 Day	3–4 Day	>5 Day	*p*-Value
Soft drinks							
Non-professional	30%	50%	10%	5%	5%	0	0.14
Professional	62.5%	29.2%	8.3%	0	0	0	
Fruit juice							
Non-professional	0	70%	20%	5%	0	5%	0.03
Professional	12.5%	29.2%	37.5%	12.5%	8.3%	0	
Isotonic/energy drink							
Non-professional	60%	15%	10%	15%	0	0	0.16
Professional	33.3%	41.7%	16.7%	8.3%	0	0	
Wine							
Non-professional	55%	35%	5%	5%	0	0	0.08
Professional	83.3%	8.3%	8.3%	0	0	0	
Beer							
Non-professional	20%	55%	20%	5%	0	0	<0.0001
Professional	91.7%	4.2%	4.2%	0	0	0	
Alcohol > 40%							
Non-professional	50%	30%	15%	5%	0	0	<0.001
Professional	95.8%	4.2%	0	0	0	0	

*p*-value indicates statistically important differences between non-professional and professional football players according to a chi-square x^2^ statistical test.

**Table 6:** Dietary habits among non-professional and professional football players.

Dietary Habits	Never/ Rarely		1–3 d Month		1–2 d Week		3–6 d Week		1 Day		>2 Day	
	N *	P *	N *	P *	N *	P *	N *	P *	N *	P *	N *	P *
White bread	15%	12.5%	15%	16.7%	20%	16.7%	35%	33.3%	15%	16.7%	0	4.2%
Cereal	5%	8.3%	20%	12.5%	35%	45.8%	20%	20.8%	15%	12.5%	5%	0
Beef **	0	0	40%	4.2%	30%	62.5%	30%	25%	0	8.3%	0	0
Burgers	0	0	20%	8.3%	35%	50%	40%	33.3%	5%	8.3%	0	0
Chicken/turkey	0	0	10%	4.2%	55%	37.5%	35%	37.5%	0	16.7%	0	4.2%
Pork	10%	8.2%	25%	16.7%	45%	50%	20%	8.3%	0	16.7%	0	0
Cold cuts	10%	8.3%	25%	25%	25%	33.3%	35%	25%	5%	8.3%	0	0
Rice/spaghetti	0	4.2%	20%	8.3%	45%	25%	35%	50%	0	8.3%	0	4.2%
Boiled potatoes	5%	4.2%	10%	37.5%	50%	29.2%	30%	20.8%	5%	8.3%	0	0
Fries **	0	45.8%	30%	37.5%	50%	4.2%	10%	8.3%	10%	0	0	4.2%
Yogurt	15%	25%	40%	41.7%	20%	25%	10%	8.3%	15%	0	0	0
Dry fruits **	45%	29.2%	5%	45.8%	40%	12.5%	10%	12.5%	0	0	0	0
Cheese	20%	33.3%	35%	25%	25%	20.8%	15%	20.8%	5%	0	0	0
Boiled eggs	20%	12.5%	20%	37.5%	25%	37.5%	35%	12.5%	0	0	0	0
Sweets	50%	66.7%	35%	29.2%	15%	4.2%	0	0	0	0	0	0
Chocolate	20%	25%	25%	54.2%	35%	12.5%	5%	4.2%	15%	4.2%	0	0
Potato Chips **	10%	54.2%	30%	41.7%	50%	4.2%	5%	0	5%	0	0	0
Olives	20%	45.8%	15%	16.7%	35%	20.8%	20%	12.5%	10%	4.2%	0	0
Virgin olive oil **	25%	12.5%	5%	25%	30%	41.7%	10%	20.8%	25%	0	5%	0
Sugar	15%	12.5%	25%	50%	30%	16.7%	10%	16.7%	15%	0	5%	4.2%

* Non-professional and professional athletes are labeled as N and P, respectively. ** indicates statistically important differences (*p* < 0.05).

**Table 7:** Total consumption of functional foods and functional drinks (*n* = 44).

Functional Foods	>6 Day	4–5 Day	2–3 Day	1 Day	5–6 d Week	3–4 d Week	1–2 d Week	2–3 d Month	1/d Month	Never/ Rarely
Fruits	0	2.3%	18.2%	31.8%	13.6%	9.1%	20.5%	2.3%	0	2.3%
Vegetables		2.3%	13.6%	20.5%	18.2%	15.9%	11.4%	9.1%	0	9.1%
Legume	0	0	2.3%	4.5%	2.3%	13.6%	52.3%	13.6%	4.5%	6.8%
Cereals	0	2.3%	4.5%	11.4%	18.2%	11.4%	18.2%	13.6%	11.4%	9.1%
Pasta	0	0	2.3%	15.9%	15.9%	27.3%	25%	2.3%	6.8%	4.5%
Dairy	0	2.3%	6.8%	18.2%	13.6%	18.2%	27.3%	6.8%	2/3%	4.5%
Fish	0	0	0	0	0	11.6%	39.5%	25.6%	11.6%	11.6%
Berries	0	0	2.3%	6.8%	2.3%	0	13.6%	18.2%	18.2%	38.6%
Pomegranates	0	0	2.3%	9.1%	2.3%	6.8%	0	13.6%	15.9%	36.4%
Fortified juices	0	0	2.3%	18.2%	2.3%	11.4%	22.7%	11.4%	6.8%	25%
Tea	0	0	0	4.5%	6.8%	9.1%	9.1%	13.6%	22.7%	34.1%
Turmeric	0	0	2.3%	0	2.3%	4.5%	2.3%	15.9%	6.8%	65.9%
Crocus sativus	0	0	0	4.5%	0	6.8%	4.5%	9.1%	11.4%	63.6%
Energy drinks *	0	2.3%	2.3%	9.3%	4.7%	14%	25.6%	11.6%	9.3%	20.9%
Coffee *	0	0	15.9%	18.2%	13.6%	15.9%	13.6%	4.5%	4.5%	13.6%
Propolis	0	0	2.3%	0	2.3%	2.3%	15.9%	9.1%	13.6%	54.5%
Honey	0	4.5%	2.3%	20.5%	11.4%	18.2%	27.3%	9.1%	2.3%	4.5%
Spirulina	0	0	0	2.3%	0	4.5%	6.8%	0	2.3%	84.1%
Soy	0	0	2.3%	0	0	2.3%	11.6%	2.3%	7%	74.45
Probiotics	2.3%	0	0	2.3%	4.5%	2.3%	13.6%	6.8%	13.6%	54.5%
Eggs *	0	0	0	16.3%	11.6%	25.6%	37.2%	9.3%	0	0

* indicates statistically important differences (*p* < 0.05).

## Data Availability

The data presented in this study are available on request from the corresponding author. The data are not publicly available due to the confidential nature of some information.
